# Establishment of a bortezomib-resistant Chinese human multiple myeloma cell line: MMLAL

**DOI:** 10.1186/1475-2867-13-122

**Published:** 2013-12-12

**Authors:** Kwan Yeung Wong, Thomas SK Wan, Chi Chiu So, Chor Sang Chim

**Affiliations:** 1 Department of Medicine, Queen Mary Hospital, University of Hong Kong, Room 419, Block K Pokfulam Road, Hong Kong, Hong Kong; 2 Department of Pathology, Queen Mary Hospital, University of Hong Kong, Hong Kong, Hong Kong

**Keywords:** Multiple myeloma, Chinese, Cell line, TP53 mutation, Isochromosome 17q

## Abstract

**Background:**

A new human myeloma cell line, MMLAL, was established from the myelomatous pleural effusion of a 73-year-old Chinese patient suffering from symptomatic International stage III IgG/lambda myeloma. After a brief period of complete remission, he developed aggressive systemic relapse complicated by malignant pleural effusion with exclusive plasma cell infiltration. His disease remained chemo-refractory, and died six months after relapse.

**Methods:**

Purified mononuclear cells from the pleural effusion of the patient were cultured in the presence of IL-6. Continually growing cells were characterized by morphological, immunophenotypic, cytogenetic, fluorescence *in situ* hybridization (FISH) and TP53 mutation analyses. Cell proliferation was measured and compared with other myeloma cell lines by cell counting at day 3, 6, 9, and 12. Drug resistance against bortezomib, a proteasome inhibitor approved as a frontline chemotherapy for eligible myeloma patients, was evaluated and compared with other myeloma cell lines by MTT assay.

**Results:**

Immunophenotypic analysis of the myeloma cells confirmed strong expression of plasma cell markers CD38 and CD138 but not T-cell or natural killer-cell marker CD56. Cytogenetic analysis of the myeloma cells showed a hypodiploid composite karyotype including loss of chromosome 13 and 17 or deletion of the short arm of chromosome 17, i.e. del(17p), in the form of isochromosome 17q10. FISH confirmed a hypodiploid karyotype with TP53 deletion but absence of t(4;14). Sequencing analysis of the TP53 gene indicated absence of mutation. Cell counting revealed that the maximum viable cell density was about 2.5 X 10^6^ cells/ml. Upon bortezomib treatment, MTT assay reported an IC_50_ of 72.17nM, suggesting a strong bortezomib resistance.

**Conclusion:**

A hypodiploid with loss of chromosome 13 and loss or del(17p) human myeloma cell line, MMLAL, was established from the pleural effusion of a Chinese myeloma patient.

## Background

Multiple myeloma is a cancer derived from malignant transformation of plasma cells [1]. It ranks the second or third most common hematological malignancy in the world. Interestingly, the incidence of myeloma in Western countries appears to be higher than that in Asian countries [2]. In the United States, the average incidence of myeloma from 2005–2009 was 5.8/100,000 [3]. By contrast, it was much lower in the Far East that it was 1.9/100,000 in Hong Kong [4], and 1.4/100,000 in Korea [5].

Clinically, myeloma arises from neoplastic transformation of a post-germinal center B cell, which will next home to the bone marrow and manifest an asymptomatic condition known as monoclonal gammopathy of undetermined significance (MGUS). MGUS will progress into symptomatic myeloma at a rate of 1% per year, associated with emergence of key end-organ damages, including hypercalcemia, renal failure, anemia and bone lesions. At the terminal stage of the disease, myeloma cells will become independent of the bone marrow stroma, resulting in the development of extramedullary myeloma such as plasma cell leukemia [6,7].

Genetically, myeloma is characterized by universal upregulation of cyclin D1, D2 or D3. However, the pathogenesis of myeloma is complicated by variable gains and losses of chromosomes that further subdivided the disease into non-hyperdiploid and hyperdiploid myeloma. Non-hyperdiploid myeloma, which represents about half of the disease, is characterized by strong association with primary immunoglobulin heavy (IgH) chain translocations, such as t(11;14)(q13;q32), t(4;14)(p16.3;q32), t(14;16)(q32;q23), t(6;14)(p21;q32) or t(14;20)(q32;q11), resulting in direct or indirect upregulation of cyclin D1, D2 or D3. On the other hand, hyperdiploid myeloma, which constitutes the other half of the disease, is usually associated with trisomies of odd-numbered chromosomes (except chr13), in particular trisomies of chr11 leads to direct upregulation of cyclin D1 [6,7].

Currently, majority of human myeloma cell lines was derived from extramedullary myeloma disease, including sacral plasmacytoma, circulating plasma cells, or myelomatous pleural effusion like our case [8]. However, there is only a few human myeloma cell lines derived from Chinese patients [9,10]. Herein, we report the establishment and characterization of a new human myeloma cell line, MMLAL, derived from a Chinese patient.

## Results

### Establishment of MMLAL

**Figure 1:**
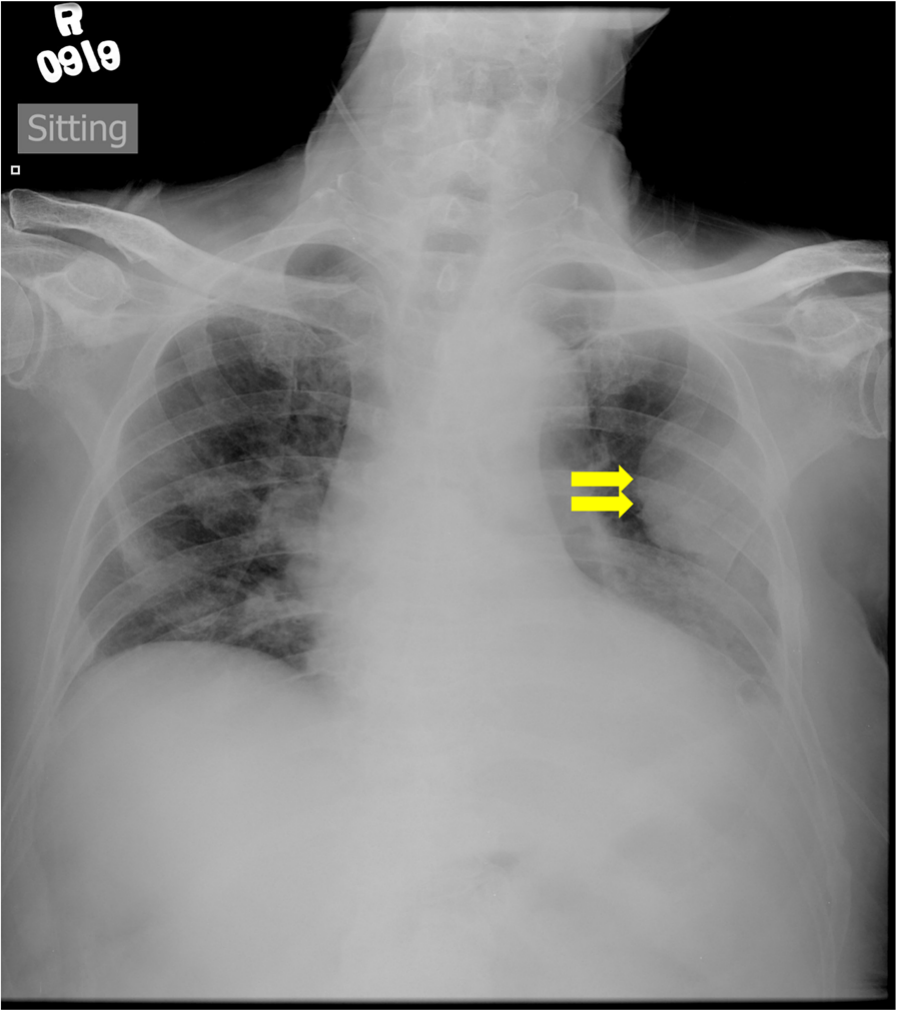
**Radiographic image of the myeloma patient.** Chest X-ray showed pleural masses after drainage of the myelomatous pleural effusion (arrows).

MMLAL was established from purified mononuclear cells, which were harvested from the pleural effusion of a Chinese myeloma patient suffering from IgG/lambda myeloma, who relapsed after a brief period of complete remission and terminated with chemo-refractory myelomatous pleural effusion (Figure 1). Cells were first cultured in a medium mixture of 40% DMEM + 40% IMDM, supplemented with rich fetal bovine serum and IL-6, an important cytokine that support myeloma cell growth in the bone marrow microenvironment. Initially, the total number of mononuclear cells decreased gradually and remained unchanged thereafter. Medium was changed in every 3 or 4 days. After 4 months, the total number of cells started to increase. The cells were cultured continuously for more than 12 months and gradually maintained in the absence of IL-6.

### Morphology of MMLAL cells

**Figure 2:**
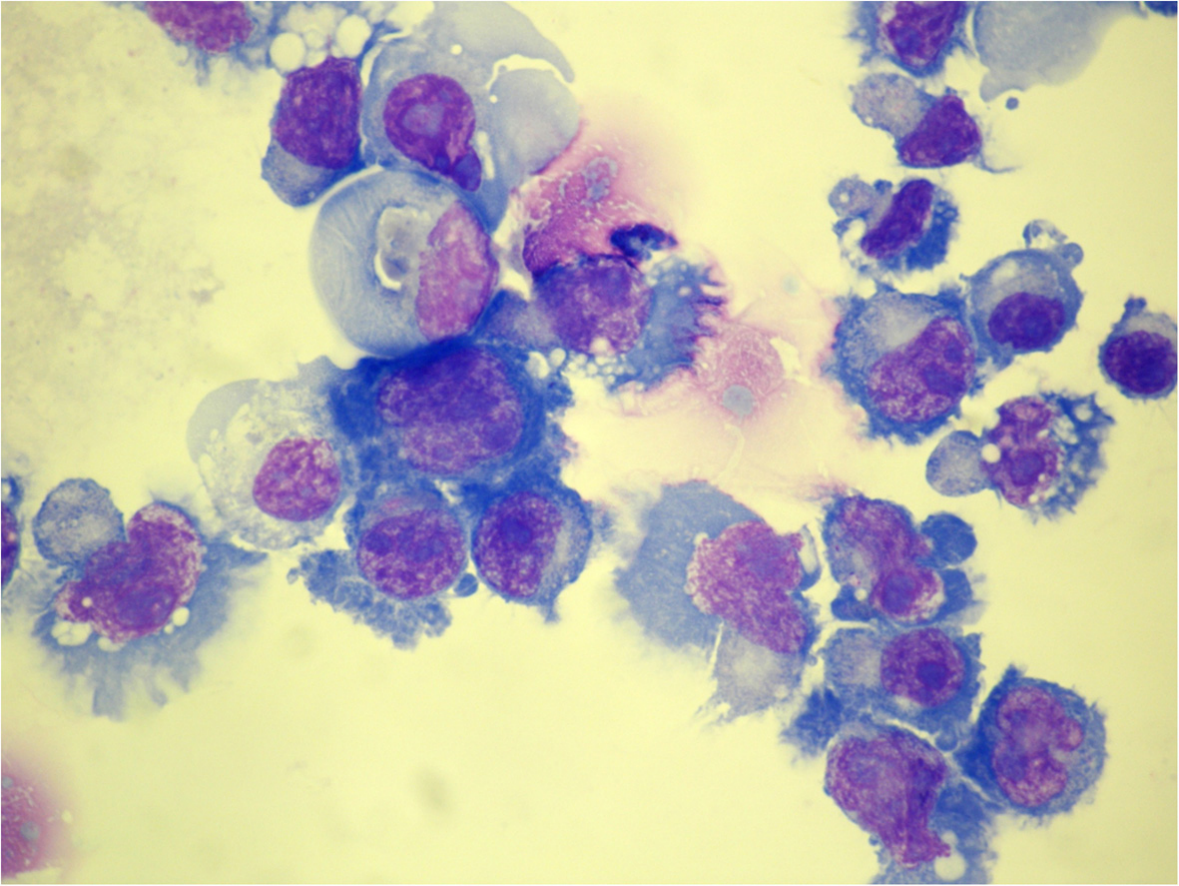
**Morphology of the MMLAL cells.** MMLAL cells were visualized with Wright’s staining, original magnification x 1000. Cells were predominantly mononuclear with eccentrically located large nuclei of oval to convoluted in shape, with condensed chromatin, prominent nucleoli, and abundant basophilic cytoplasm, with perinuclear hof.

The MMLAL cells mainly grew in suspension as clusters with a small proportion in single cells. Microphotograph of a cytospin preparation of the MMLAL cells is shown in Figure 2. The MMLAL cells were characterized by a relatively low nuclear-to-cytoplasmic ratio, eccentrically located large nuclei which are oval to convoluted in shape, prominent nucleoli, and abundant deep basophilic cytoplasm with prominent perinuclear hof.

### Immunophenotypic analysis

**Figure 3:**
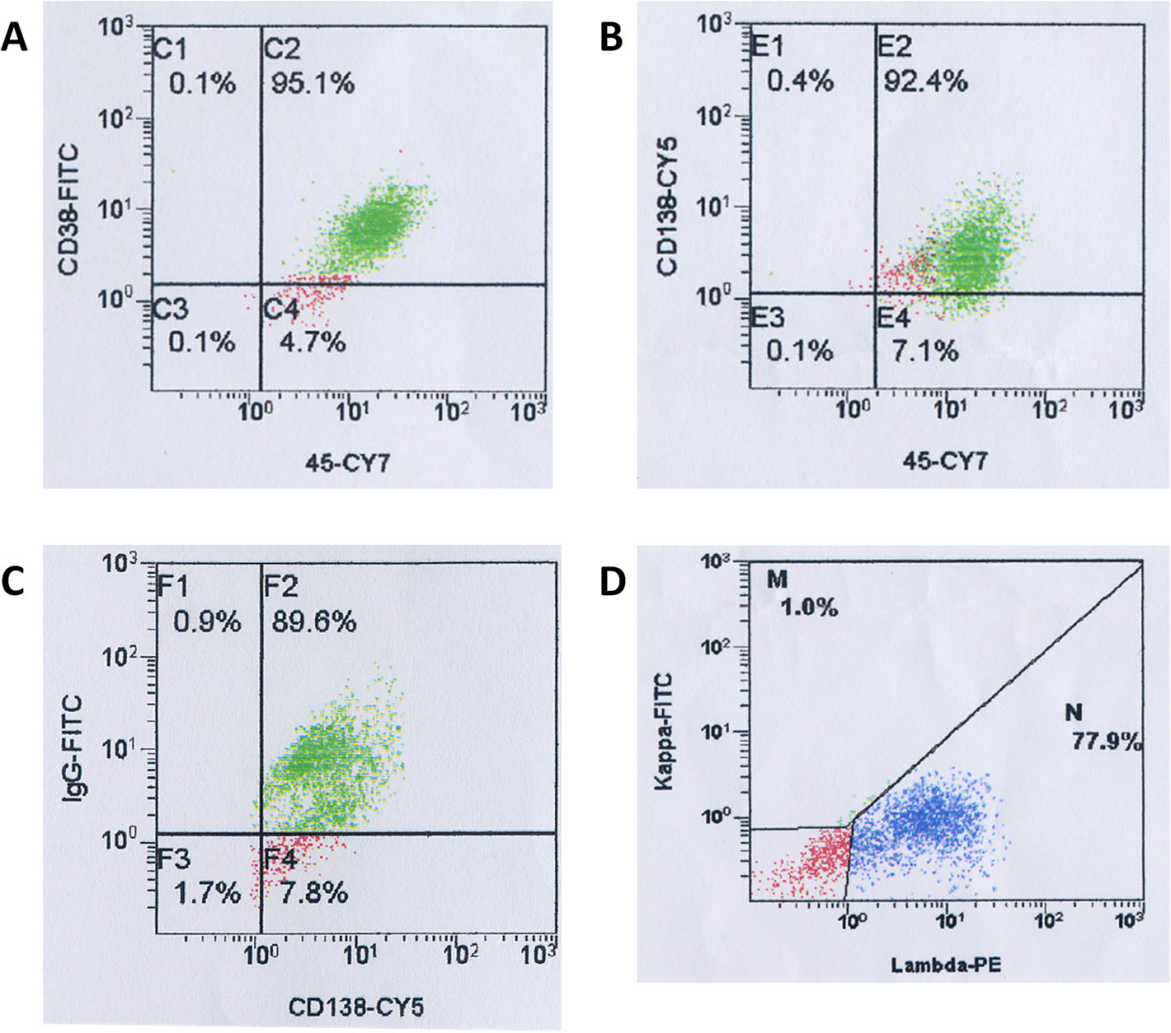
**Immunophenotypic analyses of the MMLAL cells.** The MMLAL cells were stained with **A)** anti-CD38, **B)** anti-CD138, **C)** anti-IgG, lambda, and **D)** anti-IgG, kappa. Flow cytometric analyses indicated that the MMLAL cells showed high expression of plasma cell markers CD38 and CD138, and IgG lambda light chain restriction.

Immunophenotypic analysis showed that the MMLAL cells were positive for plasma cell markers CD38 and CD138 (Figure 3A and B), and IgG lambda (Figure 3C and D), but negative for T- or NK-cell marker CD56.

### Metaphase cytogenetics

**Figure 4:**
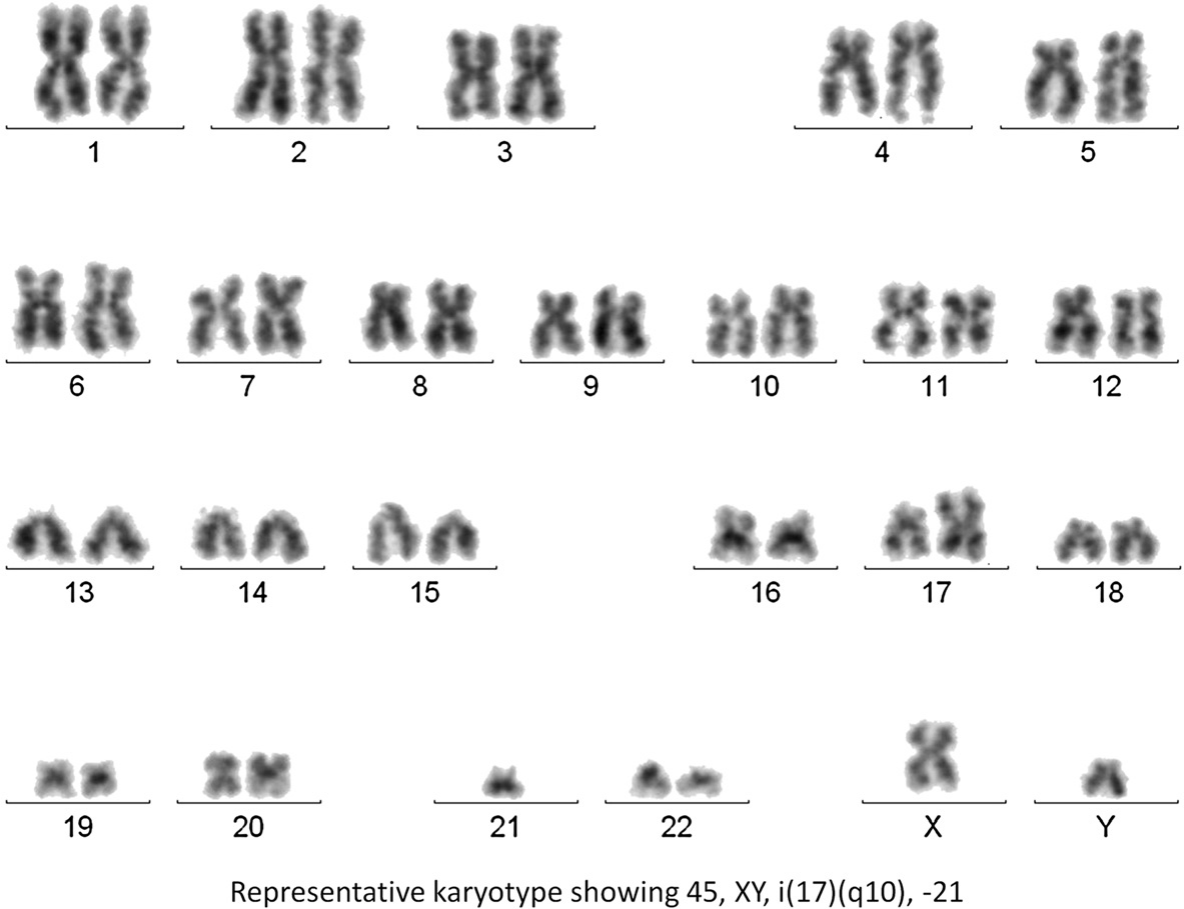
Representative karyotype of the MMLAL cells: 45, XY, i(17)(q10), -21.

Metaphase karyotyping of the MMLAL cells indicated a hypodiploid composite karyotype (chromosome number 31–46) with loss of multiple chromosomes, including chromosomes 13, 17 and 21 (Figure 4; Tables 1 and 2). Moreover, recurrent IgH translocations frequently found in myeloma that involve 14q32, such as t(11;14), t(14;16), t(6;14) or t(14;20), were not detected.

**Table 1:** MMLAL karyotype

**Cell line**	**Karyotype**
MMLAL	31 ~ 46,XY,-Y[5],-4[3],-8[3],-10[3],-13[5],-14[4],-15[3],-17[11],i(17)(q10)[2],-19[4],-20[7],-21[9],-22[6][cp19]/46,XY[5]

**Table 2:** Characteristics of the MMLAL versus other Chinese-derived (MM17 and CZ-1) and several commonly used human myeloma cell lines

**Cell line**	**MMLAL**	**MM17**	**KMS-12-PE**	**NCI-H929**	**U-266**	**LP-1**	**OPM-2**	**MOLP-8**	**CZ-1**	**RPMI8226**
**Site of origin**	Pleural effusion	Sacral plasmacytoma	Pleural effusion	Pleural effusion	Peripheral blood	Peripheral blood	Peripheral blood	Peripheral blood	Bone marrow or peripheral blood	Peripheral blood
**Gender**	XY	XX	XX	XX	XY	XX	XX	XY	XY	XY
**Ig type**	IgG lambda	IgG kappa	Ig-non-producing	IgA kappa	IgE lambda	IgG lambda	IgG lambda	IgD lambda	Light chain lambda	IgG lambda
**Ploidy**	Hypodiploid	Hypodiploid	Hypodiploid	Hypodiploid	Hypodiploid	Near-tetraploid	Near-tetraploid	Near-diploid	Hyperdiploid	Hyperdiploid
**Known IgH translocation**	Nil	t(8;14)(q24;q32)	t(11;14)(q13;q32)	t(4;14)(p16.3;q32.3)	Nil	t(4;14)(p16.3;q32.3)	t(4;14)(p16.3;q32.3)	t(11;14)(q13;q32)	Nil	t(1;14)(p13;q32)
**Chromosome 13 status**	-13	-13	del(13)(q11)	-13	-13	-13	Nil	Nil	Nil	-13
**TP53 status**	-17; wild-type	Homozygous deletion	Wild-type	Not known	Mutated	Mutated	Mutated	Not known	i(17q)	Mutated
**Reference**	[11,12]									

### Interphase FISH

**Figure 5:**
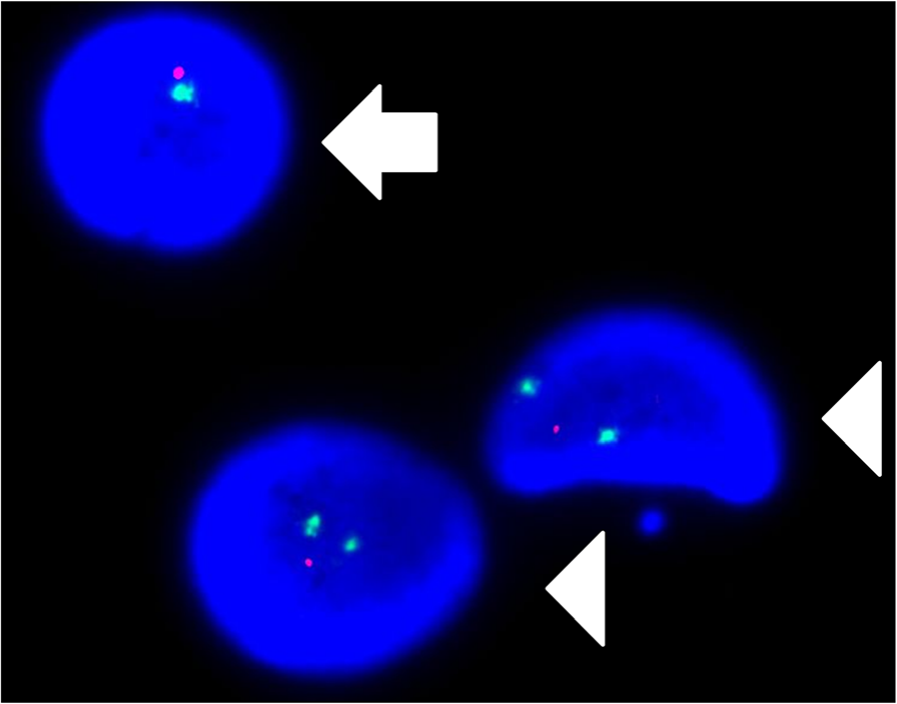
**Interphase fluorescence *in situ *hybridization (FISH) analysis of the MMLAL cells.** FISH with TP53 locus-specific (red) and chromosome 17 centromeric (green) probes indicates loss of TP53 due to monosomy 17 (arrow) and deletion 17p (arrowhead) in different nucleated cells.

FISH analyses showed that the MMLAL cells were hypodiploid with TP53 deletion (Figure 5). Moreover, FISH for t(4;14) was absent, which is cryptic and hence might be missed by metaphase cytogenetics.

### TP53 mutation analysis

No TP53 mutation was found in bidirectional sequencing analysis of exons 4–10 of TP53 of the MMLAL cells (Table 2).

### Cell growth

**Figure 6:**
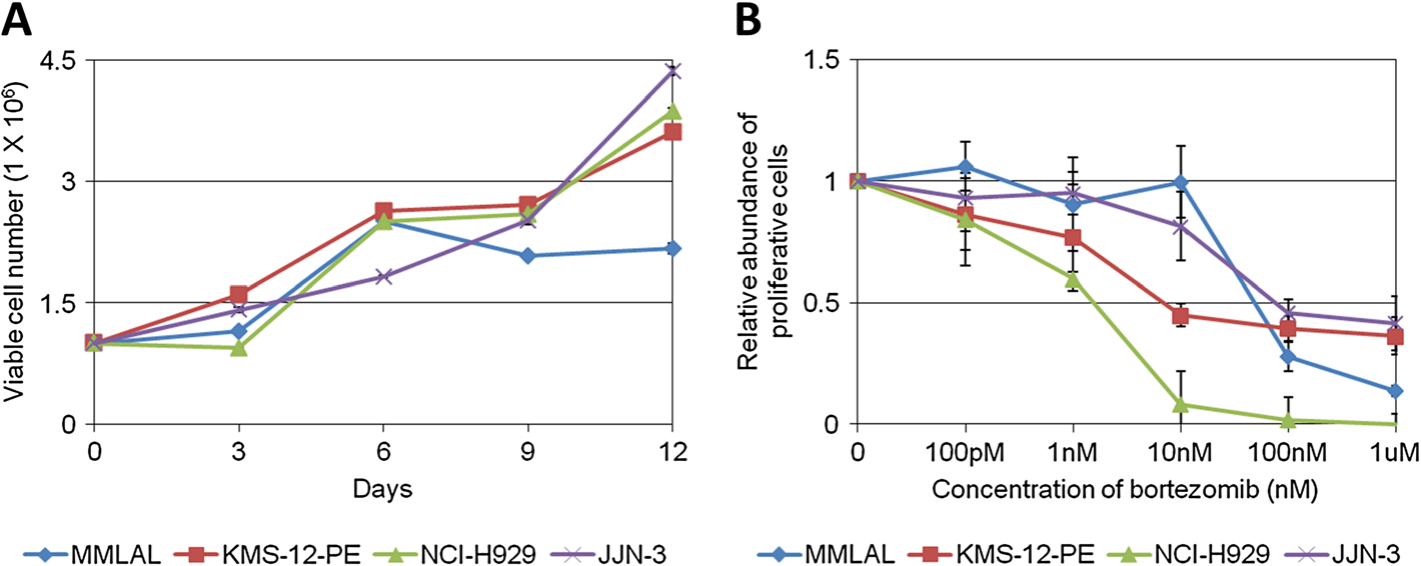
**Characterization of the MMLAL cells. A)** Cells were seeded at an initial density of 1 × 10^6^ cells/ml and allowed to grow with fresh medium change on every three days. The cells were stained by trypan blue and the number of viable cells measured by haemocytometer as indicated. **B)** Cell viability of was measured by MTT assay after treatment with different concentrations of bortezomib, and compared to the untreated control. Data were plotted by the average of three independent experiments ± standard deviations.

Counting of viable cells demonstrated that the MMLAL cells grew continuously from 1 X 10^6^ to 2.5 X 10^6^ cells/ml in 6 days, and reached a growth plateau (Figure 6A). It was in contrast to other commonly used myeloma cell lines, including KMS-12-PE, NCI-H929, and JJN-3, which might reach at a higher cell density of > 3.5 X 10^6^ cells/ml in 12 days.

### Bortezomib sensitivity

MTT assays indicated that the MMLAL cells responded to bortezomib treatment with an IC_50_ of 72.17nM, whereas other commonly used myeloma cell lines NCI-H929, KMS-12-PE, and JJN-3 showed an IC_50_ of 2.71nM, 8.55nM, and 89.39nM respectively (Figure 6B).

## Discussion

We reported the establishment of a new human myeloma cell line, MMLAL, which was derived from the pleural effusion of a Chinese patient. Based on conventional karyotyping and interphase FISH analyses, MMLAL was characterized by a hypodiploid composite karyotype, loss of chromosome 13, loss of chromosome 17 or del(17p), with the absence of primary IgH translocation and TP53 mutation.

Review of the commonly used myeloma cell lines in our laboratory together with the other two Chinese-derived myeloma cell lines, MM17 and CZ-1, most of them are hypodiploid (MM17, KMS-12-PE, NCI-H929, U-266), which are the same as the MMLAL, whereas the others are near-tetraploid (LP-1 and OPM-2), near-diploid (MOLP-8), or hyperdiploid (CZ-1 and RPMI-8226).

Despite the frequent association of hypodiploid myeloma with primary IgH translocations, MMLAL did not display any of the primary IgH translocations by conventional metaphase cytogenetics or t(4;14) by interphase FISH, which is cryptic and often missed by metaphase karyotyping [13-15]. Although a comprehensive FISH study for all known IgH translocations has not been performed, recurrent IgH translocations frequently found in myeloma that involve 14q32, such as t(11;14), t(14;16), t(6;14) or t(14;20), were not detected based in metaphase cytogenetic analysis. On the other hand, t(4;14), a cryptic translocation not detectable by metaphase cytogenetic study, has been confirmed to be negative by interphase FISH in our study. This contrasted with the presence of primary IgH translocations in other hypodiploid myeloma cell lines, including t(11;14) in KMS-12-PE, t(4;14) in NCI-H929, and t(8;14) in MM17 (Table 2). Of the other common numerical karyotypic abnormalities, MMLAL harbored concomitant loss or del(13) and del(17p). Indeed, all of these hypodiploid myeloma cell lines carry del(13). By interphase FISH, 17p loss in the MMLAL was found due to loss of the whole chromosome 17 in some cells and isochromosome 17q10 in other cells, leading to monoallelic loss of TP53. Hence, the MMLAL was found similar to most of the hypodiploid myeloma cell lines, which possesses either del(17p) or TP53 mutation, confirming that del(17p) or inactivation of TP53 is a frequent and important terminal event of the disease.

The MMLAL showed a unique growth characteristic of having a maximum cell density of 2.5 X 10^6^ cells/ml, as compared with other commonly used myeloma cell lines, including KMS-12-PE, NCI-H929, and JJN-3, which were able to proliferate at a higher cell density. While continual growing of cells has been observed after subculture with a lower cell density (data not shown), the MMLAL might be more dependent on fresh nutrient compounds or less tolerate to metabolic waste accumulated in the culture medium, than other common used myeloma cell lines. Hence, the MMLAL cells require more frequent subculture in routine culture.

Bortezomib has been widely used in the treatment of both newly diagnosed and relapsed myeloma, but drug resistance emerges as a challenge, in particular, for retreatment of relapsed myeloma [16,17]. While the IC_50_ of the MMLAL cells against bortezomib was found to be comparable to other bortezomib-resistant cell lines, which showed a resistive IC_50_ ranging from 50nM to 150nM, the MMLAL is considered to be bortezomib-resistant [18,19]. Therefore, the MMLAL may also serve as a model for elucidating the mechanism of drug resistance, or validating novel agents or approaches which may overcome the resistance.

Lastly, in addition to the lower incidence of myeloma in Chinese, there were suggestions that Chinese myeloma patients might have different profile of presenting symptoms, side-effects or outcome [2,20-22]. Therefore, availability of an additional Chinese myeloma cell line would be conducive to future mechanistic studies in case disparities in clinical presentation or outcome are verified in collaborative studies in Asia.

## Conclusion

A new Chinese-derived human myeloma cell line, characterized by hypodiploid karyotype with del(13) and loss or del(17p), was developed.

## Methods

### Clinical history

A 73-year-old man presented with severe back pain and renal failure in Dec 2009. Subsequent work-up showed that he had International stage III IgG/lambda myeloma with hypercalcemia (serum calcium: 3.89μmol/L; normal < 2.55μmol/L), multiple collapses in thoracic and lumbar vertebrae in addition to rib fractures, anemia (hemoglobin: 9.5 g/dL; normal > 11.5 g/L) and renal impairment (serum creatinine: 333 μmol/L; normal <110 μmol/L). Serum IgG measured 6510 mg/dL (normal 819-1725 mmol/L) with an IgG paraprotein of 48.9 g/L. He received melphalan, prednisolone and thalidomide achieving a complete remission in Mar 2010 but relapsed in Sep 2010 when MPT was resumed with minimal response. He was given cyclophosphamide and prednisolone since June 2011 with partial response. However, he was admitted in Dec 2011 because of left pleural effusion presenting with dyspnea. Therapeutic pleural aspiration was performed, which revealed exudative effusion with exclusively plasma cells. Moreover, chest X-ray showed a large left pleural mass (Figure 1). Chemical pleurodesis was performed, and the patient expired in Jan 2012 due to progressive myeloma. The study has been approved by Institutional Review Board of Queen Mary Hospital, and written informed consent has been obtained.

### Cell culture

Patient mononuclear cells were isolated by gradient centrifugation using Ficoll-Paque PLUS (GE Healthcare, Uppsala, Sweden), according to the manufacturer’s protocol. Initially, cells were maintained in a medium mixture of 40% DMEM + 40% IMDM, supplemented with 20% fetal bovine serum and 10 ng/ml IL-6 (R&D Systems, Minneapolis, MN, USA). When cell number started to increase, cells were gradually maintained in the medium mixture without IL-6. Medium change was done every 3 or 4 days. All media contained penicillin and streptomycin. All cells were incubated in a humidified atmosphere of 5% CO_2_ at 37°C. All cell culture reagents were obtained from Invitrogen (Carlsbad, CA, USA).

Other human myeloma cell lines included KMS-12-PE, NCI-H929, and JJN-3. KMS-12-PE was purchased from Deutsche Sammlung von Mikroorganismen und Zellkulturen GmbH (DSMZ) (Braunschweig, Germany). NCI-H929 was purchased from American Type Culture Collection (ATCC) (Manassas, VA, USA). JJN-3 was kindly provided by Dr. Wee Joo Chng (Department of Medicine, National University of Singapore). Cell lines were maintained in RPMI medium 1640, supplemented with 10-20% fetal bovine serum.

### Immunophenotypic analysis

For immunophenotyping of MMLAL cells, cells were stained with anti-CD38 (LSI19843), anti-CD138 (B-A38) (Beckman Coulter, Fullerton, USA), and anti-IgG, kappa, lambda (polyclonal) (DakoCytomation, Glostrup, Denmark). In most cases 1 × 10^4^ cells were analysed. CD138 + CD38 + CD56- cells were defined as plasma cells. Cells were acquired by FC 500 using CXP 2.2 software (Beckman Coulter).

### Cytogenetic analysis

Cell culture was fed with fresh growth medium 24 hours prior to harvest for cytogenetic study. The cells were treated with colcemid at a final concentration of 0.1 ug/mL for 1 hour at 37°C. Details of the cytogenetic techniques and slides preparation for chromosome examination were previously reported [23]. Briefly, air-dried slides were prepared following hypotonic treatment (0.075 M KCl) and acetic/methanol (1:3 v/v) fixation. Prior to banding, the slides were heated at 60°C overnight and put in 7.5% H_2_O_2_ for 3 minutes. Metaphase chromosome preparation was G-banded by 0.05% trypsin and stained with Leishman’s stain for 3 minutes. A minimum of 20 consecutive metaphases from each cell culture were fully analyzed. Metaphases were captured and karyotyped in Ikaros automated karyotyping system (MetaSystems, Germany). The karyotypes of each metaphase were described according to the International System for Human Cytogenetic Nomenclature [24].

### Interphase fluorescence in situ hybridization (FISH)

FISH was performed on slides for the examination of chromosome 13q deletions, t(4;14) and TP53 deletion by using Vysis LSI D13S319 DNA probe (13q14.3), Vysis LSI IGH/FGFR3 dual color dual fusion DNA translocation probe and Vysis LSI TP53/CEP 17 DNA probe (Abbott Molecular, IL, USA) respectively. Interphase FISH was performed on Carnoy’s fixative fixed cells according to the manufacturer’s instructions. Briefly, pre-digestion of cells on each slide was carried out in 0.05% pepsin (Sigma-Aldrich, MO, USA) in 0.01 M HCl. Subsequently, slide and probes were co-denatured by incubation at 72°C for 2 min on HYBrite hybridization system (Abbott Molecular). After incubating overnight in a dark humidity chamber at 37°C, the slide was post-hybridization washings for 2 min in 0.4x SSC/0.3% NP40 at 73°C and 30 sec in 2X SSC/0.1% NP40 at room temperature. The slide was mounted in DAPI II (Abbott Molecular, IL, USA), which contained p-phenylenediamine dihydrochloride (DAPI) as counter-stain. Subsequent image acquisition was performed using Isis FISH imaging system (MetaSystems, Germany). 200 cells were scored for the hybridization signal patterns.

### TP53 mutation analysis

Direct sequencing analysis of exons 4–10 of TP53 enables detection of most of the known TP53 mutations [25]. Genomic DNA extracted from MMLAL cells was amplified by PCR using primer sets specific to these exons, followed by bidirectional direct sequencing. Primer sets and reaction conditions were based on The International Agency for Research on Cancer (IARC) TP53 database (http://www-p53.iarc.fr) [26]. Results were compared to reference sequence NM_000546.

### Cell growth

Cells of 1 x 10^6^ were cultured with 1 ml medium in a 12-well plate, with medium change in every 3 days. The number of viable cells was recorded by trypan blue staining in every 3 days using a haemocytometer. Data from three independent experiments were plotted.

### Bortezomib sensitivity

Drug resistance against bortezomib, a frontline chemotherapeutic agent for the treatment of myeloma, was measured by 3-(4, 5-dimethylthiazolyl-2)-2, 5-diphenyltetrazolium bromide (MTT) assay. Briefly, cells were cultured with bortezomib of different concentrations, ranging from 1.0 μM to 0, for 48 hours in a 96-well plate. Each well was added with 10 μl of 5 mg/ml yellowish MTT reagent for further incubation of 4 hours, followed by addition of 100 μl of DMSO for purple formazan solubilization. Absorbance at 550 nm with reference to 650 nm was measured using an Epoch microplate spectrophotometer (Bio-Tek, Winooski, VT). After blank subtraction, result of each bortezomib concentration was obtained from average of triplicate wells and compared with that of untreated control. Data from three independent experiments with triplicate in each were plotted.

## Competing interests

We, the authors, declare that there are no competing interests.

## Authors' contributions

Conception and design: CSC; acquisition of data: KYW, TSKW, CCS; analysis and interpretation of data: all authors; writing and final approval of the manuscript: all authors.
